# Associations Between Klotho/FGF-Related Protein Expression in Peripheral Blood Mononuclear Cells, Inflammation, and Muscle Function in Middle-Aged Adults with Obesity: A Pilot Study

**DOI:** 10.3390/ijms27041983

**Published:** 2026-02-19

**Authors:** Diana G. Ariadel-Cobo, Brisamar Estébanez, Elena González-Arnáiz, María Pilar García-Pérez, Marta Rivera Viloria, Alejandra Villasante Santos, Begoña Pintor de la Maza, David Emilio Barajas-Galindo, Diana García-Sastre, María José Cuevas González, María D. Ballesteros-Pomar

**Affiliations:** 1 Institute of Biomedicine (IBIOMED), University of León, 24071 León, Spain; dgariadel@saludcastillayleon.es (D.G.A.-C.); b.estebanez@unileon.es (B.E.); egonzalezar@saludcastillayleon.es (E.G.-A.); mrivv@unileon.es (M.R.V.); avills04@estudiantes.unileon.es (A.V.S.); dballesteros@saludcastillayleon.es (M.D.B.-P.); 2 Department of Endocrinology and Nutrition, Complejo Asistencial Universitario de León (CAULE), 24071 León, Spain; pgarciapere@saludcastillayleon.es (M.P.G.-P.); bpintor.asitec@saludcastillayleon.es (B.P.d.l.M.); dabarajas@saludcastillayleon.es (D.E.B.-G.); dgarciasastre@saludcastillayleon.es (D.G.-S.)

**Keywords:** inflammaging, muscle mass, obesity, PBMCs, premature aging, sarcopenia

## Abstract

This pilot study aimed to investigate the role of the Klotho/FGF (fibroblast growth factor) system in biological features associated with premature aging, particularly inflammation and muscle dysfunction, focusing on its association with inflammatory markers, body composition, and muscle function in middle-aged adults. A total of 45 participants aged 50–60 years were enrolled, including 30 patients with obesity (22F/8M) and 15 healthy controls (11F/4M). Comprehensive assessments were conducted, including body composition analysis and muscle function tests. Evaluations of protein expression of Klotho, β-Klotho, FGF19, FGF21, FGF23, TFN-α and IL-10 were assessed in peripheral blood mononuclear cells (PBMCs). A Principal Component Analysis (PCA) was carried out to explore the relationships among variables. Significant differences were observed between the obese and control groups, with obese individuals exhibiting lower levels of Klotho and higher levels of TFN-α. The PCA revealed that higher Klotho levels were positively associated with better muscle function and lower inflammatory markers. These associations suggest that Klotho-related alterations may reflect biological processes linked to inflammation and muscle dysfunction in obesity. These findings suggest that alterations in the Klotho/FGF system may reflect biological pathways commonly associated with aging-related phenotypes in obesity, rather than direct measures of chronological aging. Given the exploratory design and limited sample size, these findings should be interpreted as hypothesis-generating rather than evidence of causal mechanisms.

## 1. Introduction

The global rise in fat-related comorbidities has positioned obesity as a major contributor to preventable mortality and a pressing public health concern [1]. Obesity is marked by metabolic dysregulation, increased oxidative stress, mitochondrial alterations, immune system impairment, and persistent low-grade inflammation [2]. Both obesity and aging involve sustained inflammatory responses and systemic dysregulation, with adipose tissue acting as a driver of metabolic impairment and inflammaging in obesity [3]. White adipose tissue may be a major contributor to inflammaging in the elderly [4,5]. The expansion of visceral and ectopic fat depots, coupled with diminished subcutaneous adipose reserves, has been identified as a central factor in promoting inflammaging [6,7]. Obesity-related changes at the cellular and molecular level mimic aging pathways, supporting the hypothesis that obesity may act as a catalyst for accelerated aging [8].

The proinflammatory environment induced by obesity accelerates sarcopenia, promoting functional decline and adverse outcomes, with immune and adipose-derived signals acting as key contributors to inflammaging [9]. One biomarker, among others, that may provide insight into the accelerated aging process and accompany obesity is Klotho [10]. Klotho has been identified as an anti-aging gene as its downregulation leads to aging-like phenotypes [11] and extends lifespan when overexpressed [12]. Human Klotho proteins are classified into α, β (KLB), and γ isoforms, with the membrane-bound form (mKL) predominantly expressed in renal tubules, the brain’s choroid plexus, and endocrine organs [13], and as a secreted protein (sKL)—the extracellular domain—that locates in the blood, urine, and cerebrospinal fluid [14,15].

Experimental studies have identified Klotho as a coreceptor for FGF23 (Fibroblast Growth Factor 23), as evidenced by the strikingly similar phenotypes observed in Klotho-deficient and FGF23-null mice, including shortened lifespan, osteoporosis/osteopenia, vascular calcification, muscle wasting, or impaired vitamin D homeostasis [16]. FGF23 and Klotho might function in a common endocrine system that regulates phosphate metabolism [17]. Under normal physiological conditions, effective FGF23 signaling depends on its interaction with a Klotho–FGFR complex [18,19]. However, sKL seems to be involved in the regulation of multiple ion channels [20,21] and growth factor receptors [15]. In addition, FGF19 and FGF21 interact with KLB [22,23]. The intestine secretes FGF19 to suppress liver bile acid synthesis in response to feeding, while the liver secretes FGF21 to promote lipolysis and other metabolic responses in white adipose tissue as a consequence of fasting [24,25]. Interestingly, FGF21 has also been found to be expressed in human muscle in response to hyperinsulinemia [26] and endocrine-acting muscle-released FGF21 leads to a browning of white adipose tissue [27]. The constant interaction between white adipose and muscle tissues, altered by age-related Klotho and FGF21 decline, may be key in sarcopenic obesity development. In this line, it has been reported that the myogenic response of muscle to mechanical loading and exercise is regulated by Klotho through genes that encode myogenin and proteins in the canonical Wnt signaling pathway [28]. Moreover, recently, it has been found that Klotho deficiency led to neuromuscular junction remodeling, myofiber denervation, and functional motor unit loss [29].

This study was designed as an exploratory, hypothesis-generating analysis. Therefore, the observed associations should not be interpreted as evidence of cause-and-effect relationships, but rather as biologically plausible links that warrant further mechanistic and longitudinal investigation.

Overall, this study aimed to investigate the role of the Klotho/FGF axis in the development of a premature aging phenotype in patients with obesity. In this context, the term “premature aging” is used to denote the presence of biological features typically associated with aging—such as chronic low-grade inflammation, impaired muscle function, and altered Klotho signaling.

## 2. Results

The mean age was 54.8 ± 0.5 years for PG and 54.4 ± 0.8 years for CG (*p* = 0.743). The mean weight was 114.3 ± 2.7 kg for PG and 63.7 ± 3.0 kg for CG (*p* < 0.0001). Patients with obesity had a mean BMI of 42.3 ± 1.0, while healthy controls had a BMI of 22.7 ± 0.6 (*p* < 0.0001). In the PG, 22 participants were female (73.3%) and 8 were male, whereas in the CG, 11 participants were female (73.3%) and 4 were male. In the PG, 11 patients (24.4%) had diabetes, 14 patients (31.1%) had high blood pressure, 12 patients (26.7%) had dyslipidemia, and 13 patients (28.9%) were active smokers. These variables are reported exclusively to describe the baseline clinical characteristics of the patient group and were not included in tabulated analyses, as they were not predefined study outcomes nor subjected to inferential statistical testing. Table 1 and Tables S1–S4 present the descriptive data of the variables analyzed (general, anthropometric, body composition, and sarcopenia-related variables) and the comparison between patient and control groups, both overall and by sex.

### 2.1. Expression Patterns of Klotho/FGF System Components in PG and CG

**Figure 1:**
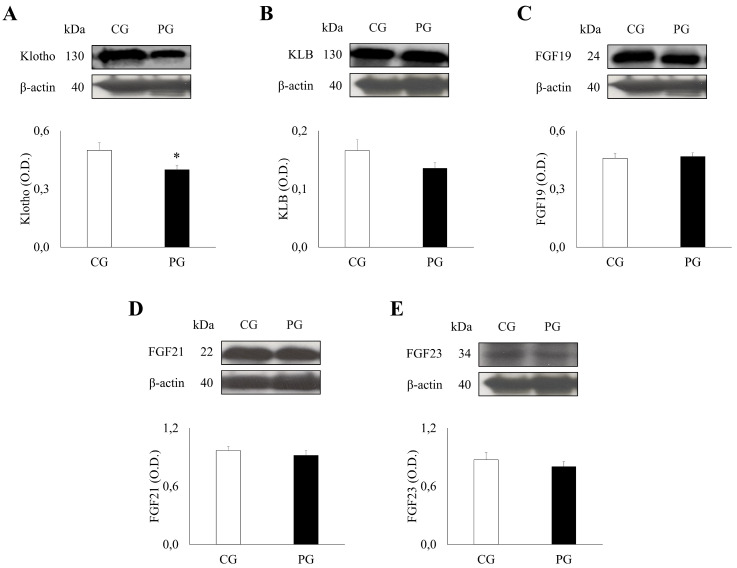
Densitometric quantification and representative Western blot of the Klotho/FGF System Components. Patients with obesity group (PG) n = 30 and control group (CG) n = 15, were analyzed for the Klotho/FGF system ((**A**), klotho, KL; (**B**), beta-klotho, KLB; (**C**), fibroblast growth factor 19, FGF19; (**D**), fibroblast growth factor 21, FGF21; and (**E**), fibroblast growth factor 23, FGF23). Mean values ± SEM are represented. Significance levels: * *p* < 0.05.

A Western blot analysis of the Klotho/FGF system revealed significant differences between PG and CG (Figure 1). Of particular interest, levels of KL were found to be significantly lower in the PG (0.40 ± 0.02) compared to the CG (0.50 ± 0.04) (*p* = 0.016). Conversely, no significant differences were observed in the levels of KLB (PG: 0.14 ± 0.01, CG: 0.17 ± 0.02, *p* = 0.115), FGF19 (PG: 0.47 ± 0.02, CG: 0.46 ± 0.02, *p* = 0.792), FGF21 (PG: 0.92 ± 0.05, CG: 0.97 ± 0.04, *p* = 0.512), and FGF23 (PG: 0.80 ± 0.05, CG: 0.87 ± 0.07, *p* = 0.438) between the groups.

### 2.2. Klotho/FGF System Component Expression Patterns in Female and Male PG Compared to CG

The Western blot analysis of the expression patterns of Klotho/FGF system components in both male and female PG compared to their respective CG was conducted. The protein levels of KL showed no significant differences between the control and PG for both females (CF: 0.48 ± 0.04, PF: 0.40 ± 0.03, *p* = 0.165) and males (CM: 0.54 ± 0.09, PM: 0.39 ± 0.03, *p* = 0.143). However, protein levels of KLB showed a significant difference between male CG and PG (CM: 0.22 ± 0.04, PM: 0.12 ± 0.02, *p* = 0.027), while no significant difference was observed in the female group (CF: 0.15 ± 0.02, PF: 0.14 ± 0.01, *p* = 0.830). Regarding the FGF family, no significant differences were found in the protein levels of FGF19, FGF21, and FGF23 for both female (FGF19: CF: 0.47 ± 0.03, PF: 0.46 ± 0.02, *p* = 0.931; FGF21: CF: 0.98 ± 0.05, PF: 0.95 ± 0.06, *p* = 0.747; FGF23: CF: 0.81 ± 0.09, 0.78 ± 0.06, *p* = 0.780) and male (FGF19: CM: 0.44 ± 0.03, PM: 0.48 ± 0.04, *p* = 0.527; FGF21: CM: 0.94 ± 0.06, PM: 0.84 ± 0.09, *p* = 0.517; FGF23: CM: 1.04 ± 0.12, PM: 0.85 ± 0.09, *p* = 0.276) groups (Table 2).

### 2.3. Expression Profiles of Pro-Inflammatory and Anti-Inflammatory Cytokines in PG and CG

**Figure 2:**
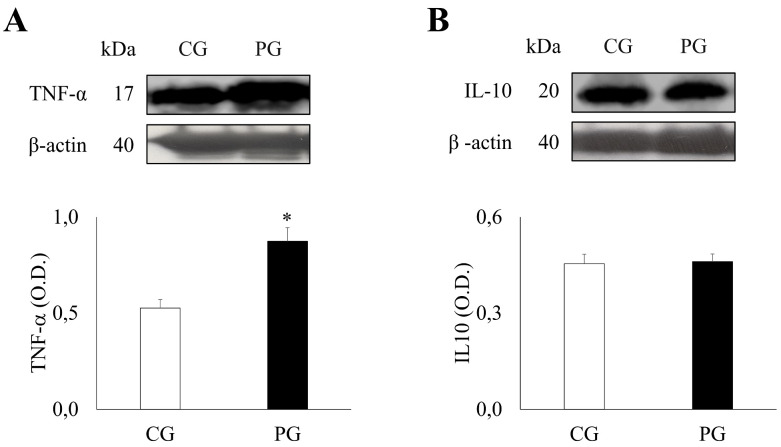
Densitometric quantification and representative Western blot of TNF-α and IL-10. Patients with obesity group (PG) n = 30 and control group (CG) n = 15, were analyzed for (**A**), TNF- α and (**B**), IL-10. Mean values ± SEM are represented. Significance levels: * *p* < 0.05.

The study of cytokine levels revealed significant differences between the PG and CG (Figure 2). Specifically, the levels of the pro-inflammatory cytokine, TNF-α, were significantly elevated in the PG (0.87 ± 0.07) compared to the CG (0.53 ± 0.04) (*p* = 0.002). Furthermore, levels of C-reactive protein (CRP), a general marker of inflammation, were significantly higher in the PG (4.52 ± 0.64) compared to the CG (0.97 ± 0.13) (*p* < 0.001) (Table S4). In contrast, levels of the anti-inflammatory cytokine, IL-10, showed no significant difference between the PG (0.46 ± 0.02) and CG (0.45 ± 0.03) groups (*p* = 0.873).

### 2.4. Expression Profiles of Pro-Inflammatory and Anti-Inflammatory Cytokines by Sex

The expression patterns of pro-inflammatory and anti-inflammatory cytokines were analyzed in both male and female PG compared to their respective control groups. The CRP biomarker showed significant differences only in females (CF: 0.91 ± 0.15, PF: 4.47 ± 0.80, *p* < 0.001) but not in males (CM: 1.13 ± 0.29, PM: 4.64 ± 1.06, *p* = 0.054). The TNF-α biomarker also showed significant differences between CG and PG in both females (CF: 0.58 ± 0.05, PF: 0.95 ± 0.09, *p* = 0.001) and males (CM: 0.39 ± 0.03, PM: 0.69 ± 0.09, *p* = 0.012). In contrast, no significant differences were observed in the levels of IL10 for both females (CF: 0.46 ± 0.04, PF: 0.47 ± 0.03, *p* = 0.782) and males (CM: 0.45 ± 0.04, PM: 0.44 ± 0.05, *p* = 0.900 (Table 3).

### 2.5. PCA by Variable Set

**Figure 3:**
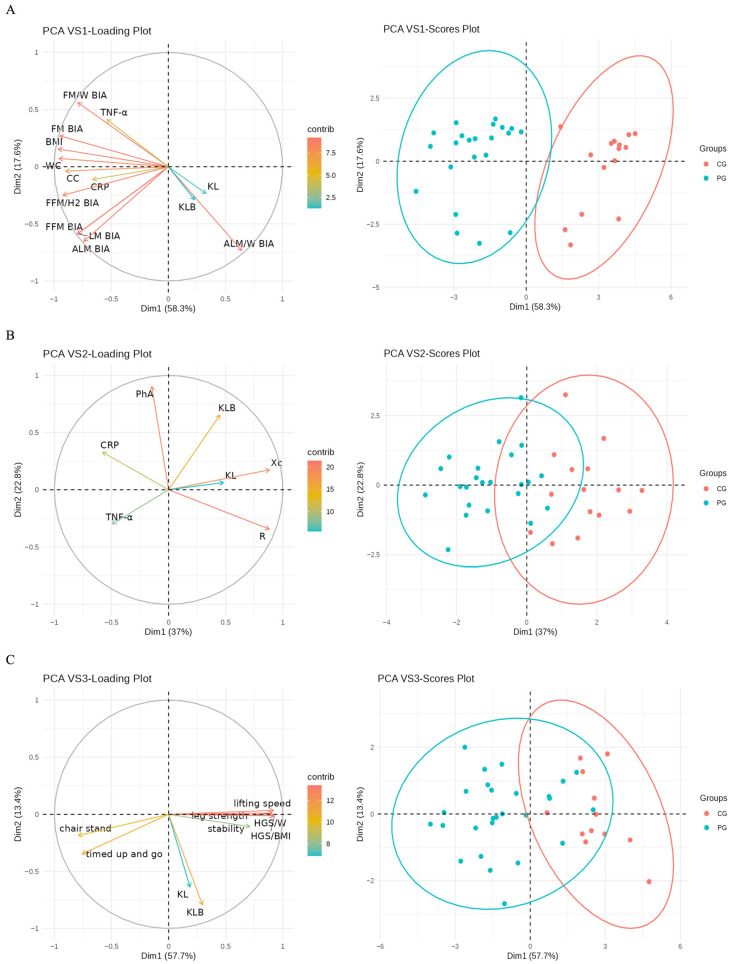
Principal Component Analysis (PCA) by Variable Set. Contribution of anthropometric, functional, and molecular variables to the principal components (PC) in the control group (CG) (Red) and the obesity group (PG) (Blue). Higher PC1 values are linked to lower BMI, less body fat, smaller circumferences, and lower inflammatory proteins, along with greater resistance and reactance. There is also a positive correlation between higher Klotho protein levels and strength, speed, and stability, with a weak negative association with lifting time. In the loading plots, arrows represent variable loadings; their direction indicates the association with the principal components and their length reflects the strength of contribution. Color intensity corresponds to the magnitude of each variable’s contribution. In the scores plots, dots represent individual subjects and ellipses indicate the 95% confidence region for each group. (**A**), Variable Set 1 (VS1); (**B**), Variable Set 2 (VS2) and (**C**), Variable Set 3 (VS3). ALM BIA, Appendicular Lean Mass by Bioimpedance; ALM/W BIA, ALM/Weight Index by Bioimpedance; BMI, Body Muscle Index; CC, Calf Circumference; CRP, C-Reactive Protein; HGS/W, Handgrip Strength/Weight Index; HGS/BMI, Handgrip Strength/Body Mass Index; FFM BIA, Fat-Free Mass by Bioimpedance; FM BIA, Fat Mass by Bioimpedance; FFM/H^2^ BIA, FFM/Height^2^ Index by Bioimpedance; FM/W BIA, FM/Weight Index by Bioimpedance; KL, Klotho; KLB, Beta Klotho; LM BIA, Lean Mass by Bioimpedance; PhA, Phase Angle; R, Resistance; TNF-α, Tumor Necrosis Factor Alpha; WC, Waist Circumference; Xc, Reactance.

In the PCA of VS1 (Figure 3A), PC1 explains approximately 58.3% of the variability, with positive loadings of ALM/W BIA (0.23), KL (0.12), and KLB (0.08), and negative loadings of BMI (−0.34), FM BIA (−0.33), FFM (−0.28), ALM BIA (−0.26), WC (−0.34), CC (−0.32), TNF-α (−0.19), and CRP (−0.23). Moreover, PC2 explains around 17.6% of the variability, with positive loadings of TNF-α (0.26), FM/W BIA (0.36), and FM BIA (0.17), and negative loadings of FFM BIA (−0.38), LM BIA (−0.38), ALM BIA (−0.42), ALM/W BIA (−0.47), and FFM/H2 BIA (−0.16).

Thus, subjects with a high PC1 value have lower BMI, reduced body fat, smaller circumferences, lower inflammatory proteins, and higher muscle mass, along with elevated Klotho levels. PC2 is linked to inflammation, where higher values correspond to increased TNF-α and fat, with lower fat-free mass, lean mass, and ALM.

In the PCA of VS2 (Figure 3B), PC1 explains approximately 37% of the variability, with positive loadings of Resistance (0.88), Reactance (0.88), KL (0.49), and KLB (0.45), and negative loadings of TNF-α (−0.50), and CRP (−0.58). Moreover, PC2 explains approximately 22.80% of the variability, with positive loadings of Phase Angle (0.90), KLB (0.65), and CRP (0.33), and negative loadings of Resistance (−0.34) and TNF-α (−0.30).

Thus, subjects with high PC1 values exhibit greater resistance and reactance, elevated levels of Klotho proteins, and reduced levels of inflammatory markers.

In the PCA of VS3 (Figure 3C), PC1 explains approximately 57.7% of the variability, with positive loadings of HGS/W (0.40), HGS/BMI (0.41), Strength Legs (0.40), Speed Lifted (0.40), stability (0.31), KL (0.08), and KLB (0.13), and negative loadings of Chair Stand (0.35) and Test Up Go (−0.33). Moreover, PC2 explains approximately 13.40% of the variability, with positive loadings of Strength Legs (0.01), and negative loadings of Chair Stand (−0.17), Test Up Go (−0.32), Stability (−0.10), KL (−0.58), and KLB (−0.72).

Thus, higher levels of Klotho proteins correlate positively with strength, speed, and stability, and negatively with lifting time in PC1. PC2 indicates that lower Klotho levels are associated with shorter lifting times and reduced stability.

The ANOVA showed significant differences of the three PC1 derived from the groups of variables between the groups (*p* < 0.001).

The third principal component (PC3) explained a substantially lower proportion of the total variance across all variable sets and did not reveal a clear or biologically interpretable pattern between groups. Therefore, PC3 was not further explored in the Results Section.

## 3. Discussion

Due to the limited research on biomarkers linking obesity to early aging, this pilot study offers valuable initial insights and justifies the need for a larger sample to confirm these preliminary observations.

Participants aged 50 to 60 years were selected because this life stage represents a transitional period in which, if obesity is associated with biological processes linked to aging, such alterations may be more detectable than in younger individuals (who may not yet exhibit these features) and more interpretable than in older adults, in whom aging-related changes are already established. This age range also minimizes potential hormonal confounding, as all female participants were postmenopausal. A notable strength of this study is the comprehensive assessment of body composition and muscle performance, which complements the biochemical analyses and allows for an exploratory evaluation of the relationship between obesity, inflammation, and muscle dysfunction. Although this study does not directly assess biological aging through established aging biomarkers or longitudinal outcomes, the observed inflammatory, muscular, and molecular alterations are consistent with biological processes commonly associated with aging, providing a relevant biological framework for interpreting the findings.

The study identified lower KL expression in PBMCs among participants with obesity compared to controls, pointing toward a potential obesity-associated downregulation. Literature has reported conflicting results regarding the association between obesity and serum klotho levels. Indeed, sedentary middle-aged subjects with obesity had higher serum klotho levels than normal weight group [30]. Likewise, increased serum klotho levels were reported among children and adolescents with obesity hospitalized with gastrointestinal symptoms [31]. The observed decline in KL levels among individuals with obesity may indicate a broader impairment of Klotho-related physiological pathways, including decreased systemic availability. This observation aligns with earlier reports showing lower sKL levels in populations with obesity, particularly among women and those with increased visceral fat. Similarly, Cheng et al. identified a negative correlation between sKL concentrations and the presence of metabolic syndrome, notably with central adiposity and hypertriglyceridemia [32]. On the other hand, obesity among women was significantly and inversely associated with serum klotho levels. Similarly, women who developed obesity during their lifetime had consistently lower Klotho levels than their never-obese counterparts [10]. Other research also demonstrated that serum Klotho levels were negatively correlated with waist circumference in young women with polycystic ovary syndrome and among community-dwelling adults with abdominal obesity [32,33]. Furthermore, young Japanese women with restrictive-type anorexia nervosa or obesity had significantly lower serum klotho levels than those with a healthy body weight [34]. Similar findings have been reported in studies involving lifestyle modifications. In these cases, weight reduction—achieved with or without exercise—was associated with elevated circulating Klotho levels in adults with overweight or obesity. Notably, the rise in Klotho was inversely correlated with reductions in body weight, BMI, fat mass, and waist circumference [35]. On the other hand, aerobic training increases renal Klotho and decreases FGF23 in obese Zucker rats, thus combating the imbalance caused by obesity [36].

In contrast, KLB levels did not differ significantly. Unlike KL, KLB exists solely as a membrane-bound form, predominantly expressed in the liver and white adipose tissue, and is absent from circulation. Among Klotho isoforms, only sKL circulates systemically and acts as an anti-aging hormone independently of FGF23, without functioning as a co-receptor [37].

Regarding the FGF family (FGF 19, 21 and 23), the absence of differences could also be related to the sample size. Although Hu et al. [38] showed that serum FGF23 levels are elevated in both men and postmenopausal women with obesity, especially those with abdominal obesity, other authors highlighted the possibility of independence of Klotho to FGF families, as aforementioned. The assessment of Klotho expression in PBMCs provides insight into the immunometabolic component of obesity-related inflammation. Beyond its classical endocrine role, Klotho has been shown to exert anti-inflammatory and antioxidant effects in immune cells, including the modulation of NF-κB signaling, suppression of pro-inflammatory cytokine production, and regulation of oxidative stress pathways [32]. Therefore, PBMC-derived Klotho expression may reflect systemic inflammatory burden and immune dysregulation associated with obesity; however, the absence of circulating Klotho measurements represents a limitation of the present study.

The reduced Klotho levels observed in individuals with obesity may reflect a link between obesity and biological processes commonly associated with aging. The absence of significant differences in other components of the Klotho/FGF system suggests that Klotho-related alterations may represent one of several biological pathways involved in obesity-associated inflammation and muscle dysfunction. These findings do not support the superiority of Klotho over established clinical markers, but rather point to its potential complementary role within a broader biological framework. Overall, no statistically significant sex-related differences were observed in most components of the Klotho/FGF system. These results should be interpreted with caution, given that this is a preliminary study.

Adiposity itself is an active endocrine organ, secreting adipokines that result in an unnatural environment promoting free radicals and inflammation. Increased free radical production and inflammation are interrelated, and unfortunately, adiposity stimulates both detrimental factors [39]. Oxidation of lipoproteins induces monocytes to release pro-inflammatory cytokines. Along with the oxidized lipoprotein, adipose tissue itself secretes the inflammatory cytokines IL-6 and TNF-α, resulting in elevated levels in the patient with obesity [39]. As anticipated, proinflammatory markers like TNF-α and CRP showed notable differences. TNF-α, a key mediator of adipose tissue inflammation, has been shown to downregulate Klotho expression and interfere with FGF21 signaling in adipocytes [40]. The present findings strongly support the former possibility, evidencing that the enhanced inflammation in PBMCs in obesity, exemplified by increased TNF-α levels, leads to strong downregulation of Klotho protein levels.

Moreover, our results suggest that the expression profiles of pro-inflammatory and anti-inflammatory cytokines may differ between males and females with obesity, highlighting the importance of considering sex as a factor in inflammation-related studies. Further investigations are necessary to explore the potential implications of these cytokine biomarkers in the development and management of obesity.

The PCA showed that lower body weight, accompanied by higher muscle mass, strength, and muscle performance, is associated with elevated levels of Klotho. Conversely, higher body fat percentage and lower muscle mass are linked to increased levels of inflammatory markers such as TNF-α. These findings align with previous studies, reinforcing the relevance of Klotho within biological processes commonly associated with aging.

In addition to the limitation related to the sample size, it is important to emphasize that there is currently no available literature specifically addressing the regulation of the Klotho/FGF axis in PBMCs. Consequently, the interpretation of the present findings has been guided by existing evidence derived from other tissues and biological compartments, primarily adipose tissue, kidney, and serum. Accordingly, in this study, premature aging is used as a conceptual framework integrating inflammation and muscle dysfunction, rather than as a definitive or direct measure of aging per se.

Within this immunometabolic framework, peripheral blood mononuclear cells are closely involved in immune aging processes. Immunosenescence, characterized by functional and phenotypic changes in immune cells, has been associated with chronic low-grade in-flammation and metabolic alterations [41]. Although the present study does not directly assess immunosenescence, PBMC-based analyses may capture aspects of these systemic aging-related processes.

However, this approach also represents a strength, as it opens a novel perspective on the immunological dimension of Klotho signaling in obesity and supports future research aimed at integrating immune, metabolic, and functional biomarkers of aging-related phenotypes.

## 4. Materials and Methods

### 4.1. Participants and Ethical Approval

Forty-five individuals, consisting of men and postmenopausal women aged between 50 and 60 years, were included in this study. Thirty patients with obesity (PG, 22F/8M, diagnosed with central obesity with a body mass index (BMI) greater than or equal to 30 kg/m^2^ and a waist circumference equal to or greater than 102 cm in men and 88 cm in women) and fifteen healthy control subjects (CG, 11F/4M, BMI less than 30 kg/m^2^ and a waist circumference of less than 102 cm in men and less than 88 cm in women), matched by age and gender, were selected and recruited from the Endocrinology clinics at the Complejo Asistencial Universitario de León (CAULE) to participate. No sex-based stratification was applied during recruitment. The exclusion criteria, which were applied equally to both the patient and control groups, included premenopausal women, kidney disease with a glomerular filtration rate below 60 mL/min, liver disease with plasma AST, ALT, or GGT levels greater than twice the upper limit of normal, active cancer, or cardiac or respiratory failure requiring pharmacological treatment. The presence of metabolic comorbidities (type 2 diabetes, hypertension, dyslipidemia) and smoking status was recorded exclusively for baseline clinical characterization of the study population and was not included as predefined study outcomes or subjected to inferential statistical analysis. This study followed the principles of the Declaration of Helsinki, and all patients provided written informed consent. The study protocol has been registered at ClinicalTrials.gov as an observational study, with the assigned number NCT05443711, and it was approved by the Clinical Research Ethics Committee of the Complejo Asistencial Universitario de León on 9/7/2021 (Internal Registration No: 21125).

Body Composition: Both the PG and CG underwent anthropometric measurements (weight, height, BMI, waist and calf circumferences) and bioimpedance analysis (BIA, TANITA MC-780A; TANITA, Tokyo, Japan), including phase angle and estimation of fat mass, fat-free mass, and appendicular muscle mass. In addition, in the PG, body composition was assessed by Dual X-ray Absorptiometry (DXA, Lunar iDXA, GE Healthcare, Chicago, IL, USA), obtaining values for bone mass, fat mass (FM), fat-free mass (FFM), and appendicular lean mass (ALM). In both groups, muscle strength was assessed by hand grip strength using the JAMAR dynamometer and the 5-times chair stand test. Muscle function was also assessed using the TANITA BM-220 force plate (BM-220; TANITA Co., Ltd., Tokyo, Japan), which assesses muscle strength, balance, and standing speed, along with the Timed Up and Go test.

Biochemical Parameters Assessment: Biochemical parameters were evaluated according to the clinical protocol of our clinic: blood glucose, insulin, Homeostatic Model Assessment of Insulin Resistance (HOMA-IR = [Fasting Glucose (mg/dL) Fasting Insulin (µU/mL)]/405), renal function (creatinine, glomerular filtration rate), and liver function (Aspartate Aminotransferase-AST-, Alanine Aminotransferase-ALT-, and Gamma-Glutamyl Transferase—GGT), lipid profile (total cholesterol, HDL, and LDL), bone metabolism parameters (calcium, phosphorus, magnesium, parathyroid hormone-PTH, 25OH vitamin D, osteocalcin, beta crosslaps), albumin, prealbumin, retinol-binding protein, and C-reactive protein.

### 4.2. Blood Sampling

Venous blood samples (30 mL) were obtained from the antecubital vein of volunteers using the BD Vacutainer™ system containing ethylenediamine tetraacetic acid (EDTA) as the anticoagulant (BD, Franklin Lakes, NJ, USA). All samples were collected between 09:00 a.m. and 10:00 a.m. following an overnight fast. The blood samples were centrifuged at 1300× *g* (3000 rpm) for 10 min at 4 °C, and the plasma was stored at −80 °C until further analysis.

#### Isolation of Peripheral Blood Mononuclear Cells (PBMCs)

The cellular fraction of the blood was layered over a Ficoll separation solution (Biochrom AG, Berlin, Germany) and centrifuged at 240× *g* (1200 rpm) for 40 min at room temperature. The PBMC interface and pellet were washed in phosphate-buffered saline (PBS), pH 7.4, and recovered by two sequential centrifugations at 890× *g* (2300 rpm) for 10 min and 500× *g* (2400 rpm) for 5 min, both at room temperature. The samples were then stored at −80 °C until further analysis.

The use of PBMCs was chosen as a minimally invasive and ethically acceptable approach, particularly relevant in middle-aged and older adults with obesity. The collection of skeletal muscle or adipose tissue biopsies in this population is often limited by ethical concerns, patient burden, and feasibility constraints in non-interventional clinical settings. Therefore, PBMCs represent a pragmatic biological compartment that allows the investigation of immunometabolic pathways potentially involved in obesity-related inflammation and functional decline.

### 4.3. Western Blot Analysis

PBMC lysis was performed in a pH 7.4 buffer consisting of 10 mM Tris, 1 mM EDTA, and 0.25 mM sucrose, to which protease (cOmplete™ Mini EDTA-free Protease Inhibitor Cocktail, Roche, Switzerland) and phosphatase (Sigma-Aldrich, St. Louis, MO, USA) inhibitor cocktails were added. Exosomes were lysed with a pH 7.4 RIPA buffer, consisting of 50 mM Tris HCl, 150 mM KCl, 0.5% sodium deoxycholate, 0.1% SDS (sodium dodecyl sulfate), and 1% NP-40 (Nonidet P-40), to which the protease inhibitor cocktail was added (cOmplete™ Mini EDTA-free Protease Inhibitor Cocktail, Roche, Switzerland).

The Bio-Rad Protein Assay (Cat# 5000006, Bio-Rad, Hercules, CA, USA) was used to measure the protein content of PBMC and exosome samples in the microplate reader Synergy H1 (BioTek, Winooski, VT, USA). Fifteen µL of exosome samples and PBMC samples containing 20 µg of PBMC protein were fractionated by SDS-PAGE on 13% polyacrylamide gels. Separated proteins were transferred to polyvinylidene difluoride (PVDF) membranes and pre-incubated in 5% non-fat milk for 60 min at room temperature to block non-specific binding.

Afterward, membranes were incubated overnight at 4 °C with specific primary antibodies. Antibodies against FGF-19 (Cat# sc-390621), FGF-21 (Cat# sc-518101), IL-10 (Cat# sc-7888, RRID: AB_2125230), α-Klotho (Cat# sc-515942), and TNF-α (Cat# sc-1348, RRID: AB_632513) were purchased from Santa Cruz Biotechnology (Dallas, TX, USA). Antibodies against FGF-23 (Cat# MAB2629, RRID: AB_2247024) and KLB (Cat# AF5889) were purchased from R&D Systems (Bio-Techne, Minneapolis, MN, USA). Bound primary antibody was detected using horseradish peroxidase (HRP)-conjugated secondary antibody (Dako, Glostrup, Denmark) along with enhanced chemiluminescence (ECL) Western Blotting Substrate (Pierce™, Thermo Scientific™, Waltham, MA, USA), and blot signals were captured with an Odyssey^®^ XF Imaging System (LI-COR^®^, Lincoln, NE, USA) and its LI-COR^®^ Acquisition Software version 2.0 (LI-COR^®^, Lincoln, NE, USA). Finally, the optical density (O.D.) of the specific bands was quantified with an imaging densitometer (Image J, Bethesda, MD, USA), and densitometric analyses of PBMCs were normalized to β-actin (Cat# A3854, RRID: AB_262011, Sigma-Aldrich, Munich, Germany).

### 4.4. Statistical Analysis

The study data were analyzed using R v4.2.2 software with a *p* < 0.05 as the limit of statistical significance. Outlier values were detected by the Inter-Quartile Range (IQR) and replaced by the median using the dplyr R package. Descriptive analyses were used to examine data using the psych R package. Shapiro–Wilk tests were used to test the normality of the variables. F-test was used to test homoscedasticity (homogeneity of variances). Differences between groups were examined using a *t*-test for normally distributed variables and a Mann–Whitney U test for non-normally distributed variables. Sex-by-group effects were analyzed using two-way ANOVA or Kruskal–Wallis test, depending on the nature of the variables.

Principal Component Analysis (PCA) was used to explore multivariate patterns and relationships among anthropometric, body composition, inflammatory, and functional variables, and to assess differences between groups, rather than as a confirmatory or predictive statistical model. PCA was performed using three predefined sets of variables: Variable Set 1 (VS1): BMI, FM BIA, FFM BIA, LM (Lean Mass) BIA, ALM BIA, ALM/W BIA, FM/W BIA, FFM/H^2^ BIA, WC (Waist Circumference), CC (Calf Circumference), KL, KLB, TNF-α, and CRP; Variable Set 2 (VS2): phase angle, resistance, reactance, KL, KLB, TNF-α, and CRP; and Variable Set 3 (VS3): HGS/W (Hand Grip Strength-to-Weight ratio), HGS/BMI (Hand Grip Strength-to-Body Mass Index ratio), chair stand, timed up and go, leg strength, lifting speed, stability, KL, and KLB. PCA was conducted to evaluate the contribution of these variables to the principal components (PC1, PC2, and PC3). Group differences in the principal components were assessed using analysis of variance (ANOVA). Although three principal components were extracted, only components with clear biological interpretability and relevant explained variance were retained for detailed analysis.

## 5. Conclusions

Reduced Klotho levels in individuals with obesity are associated with inflammatory and functional alterations commonly linked to aging-related biological processes. This reduction is associated with higher inflammatory markers, suggesting that obesity-driven inflammation may be linked to biological processes commonly associated with premature aging. Higher Klotho levels correlate with improved muscle performance. Klotho may represent a biologically relevant marker associated with inflammatory and functional alterations commonly linked to aging-related processes in obesity. However, its clinical utility cannot be inferred from the present pilot study.

## Figures and Tables

**Table 1:** General and Anthropometric Data.

	CG (n = 15)	PG (n = 30)	*p*-Value
	Mean ± SEM	Mean ± SEM	PG vs. CG
Age (years)	54.4 ± 0.76	54.77 ± 0.54	0.743
Weight (Kg)	63.73 ± 3.01	114.27 ± 2.71	0.000 ***
Height (m)	1.67 ± 0.02	1.65 ± 0.01	0.411
BMI (kg/m^2^)	22.73 ± 0.55	42.34 ± 0.97	0.000 ***
WC (cm)	85.20 ± 2.24	125.86 ± 2.29	0.000 ***
CC (cm)	35.04 ± 0.68	45.35 ± 0.69	0.000 ***

BMI, Body Mass Index; CC, Calf circumference; CG, control group; PG, patient group; SEM, standard error of the mean; WC, Waist circumference. *** *p* < 0.001.

**Table 2:** Klotho/FGF system expression levels in female and male CG and PG.

	CF	CM	PF	PM	*p*-Values				
	Mean ± SEM	Mean ± SEM	Mean ± SEM	Mean ± SEM	Group * Sex	CF vs. PF	CM vs. PM	CF vs. CM	PF vs. PM
KL (O.D.)	0.48 ± 0.04	0.54 ± 0.09	0.40 ± 0.03	0.39 ± 0.03	0.252	0.165	0.143	0.744	0.964
KLB (O.D.)	0.15 ± 0.02	0.22 ± 0.04	0.14 ± 0.01	0.12 ± 0.02	0.008 **	0.830	0.027 *	0.102	0.242
FGF19 (O.D.)	0.47 ± 0.03	0.44 ± 0.03	0.46 ± 0.02	0.48 ± 0.04	0.435	0.931	0.527	0.609	0.738
FGF21 (O.D.)	0.98 ± 0.05	0.94 ± 0.06	0.95 ± 0.06	0.84 ± 0.09	0.771	0.747	0.517	0.664	0.326
FGF23 (O.D.)	0.81 ± 0.09	1.04 ± 0.12	0.78 ± 0.06	0.85 ± 0.09	0.358	0.780	0.276	0.186	0.550

CF, control female; CM, control male; FGF, fibroblast growth factor; KL, klotho; KLB, beta-klotho; O.D., optical density; PF, patient female; PM, patient male; SEM, standard error of the mean. ** *p* < 0.01; * *p* < 0.05.

**Table 3:** Inflammatory proteins expression levels in female and male control and patients with obesity.

	CF	CM	PF	PM	*p-*Values				
	Mean ± SEM	Mean ± SEM	Mean ± SEM	Mean ± SEM	Group * Sex	CF vs. PF	CM vs. PM	CF vs. CM	PF vs. PM
CRP (mg/L)	0.91 ± 0.15	1.13 ± 0.29	4.47 ± 0.80	4.64 ± 1.06	0.001 **	0.001 ***	0.054.	0.448	0.572
IL-10 (O.D.)	0.46 ± 0.04	0.45 ± 0.04	0.47 ± 0.03	0.44 ± 0.05	0.604	0.782	0.900	0.948	0.603
TNF-α (O.D.)	0.58 ± 0.05	0.39 ± 0.03	0.95 ± 0.09	0.69 ± 0.09	0.929	0.001 ***	0.012 *	0.062.	0.082.

CF, control female; CM, control male; CRP, C-reactive protein; IL, interleukin; TNF, tumoral necrosis factor; PF, patient female; PM, patient male; SEM, standard error of the mean. *** *p* < 0.001; ** *p* < 0.01; * *p* < 0.05.

## Data Availability

Data is unavailable due to privacy or ethical restrictions.

## Supplementary Materials

The following supporting information can be downloaded at: https://www.mdpi.com/article/10.3390/ijms27041983/s1.
