# Supplementary material for: Associations Between Klotho/FGF-Related Protein Expression in Peripheral Blood Mononuclear Cells, Inflammation, and Muscle Function in Middle-Aged Adults with Obesity: A Pilot Study
Source: Int J Mol Sci. 2026 Feb 19;27(4):1983. doi: 10.3390/ijms27041983 (PMC12940495; doi:10.3390/ijms27041983)
Supplement: Supplementary file 1 [file ijms-27-01983-s001.zip › ijms-4101279-supplementary.pdf]

Supporting Information

Table S1. Body composition (Bioimpedance analysis TANITA MC 780).

	CG	PG	p-value	CF	CM	PF	PM	p-values				
	Mean ± SEM	Mean ± SEM	PG vs. CG	Mean ± SEM	Mean ± SEM	Mean ± SEM	Mean ± SEM	Group*Sex	CF vs. PF	CM vs. PM	CF vs. CM	PF vs. PM
**Age (years)**	54.4 ± 0.76	54.77 ± 0.54	0.743	54.82 ± 0.89	53.50 ± 1.55	54.48 ± 0.63	55.44 ± 1.08	0.643	0.734	0.311	0.470	0.349
**Weight (Kg)**	63.73 ± 3.01	114.27 ± 2.71	0.000***	58.89 ± 2.46	77.03 ± 4.79	110.04 ± 2.97	124.15 ± 4.43	0.000***	0.000***	0.007**	0.016*	0.016*
**Height (m)**	1.67 ± 0.02	1.65 ± 0.01	0.411	1.63 ± 0.02	1.76 ± 0.03	1.62 ± 0.01	1.71 ± 0.02	0.363	0.639	0.160	0.008**	0.000***
**BMI (kg/m²)**	22.73 ± 0.55	42.34 ± 0.97	0.000***	22.03 ± 0.56	24.68 ± 0.85	41.93 ± 1.02	43.30 ± 2.25	0.737	0.000***	0.000***	0.027*	0.525
**WC (cm)**	85.20 ± 2.24	125.86 ± 2.29	0.000***	83.91 ± 2.54	88.75 ± 4.77	122.89 ± 2.17	132.80 ± 5.24	0.488	0.000***	0.000***	0.358	0.045*
**CC (cm)**	35.04 ± 0.68	45.35 ± 0.69	0.000***	34.59 ± 0.78	36.28 ± 1.31	45.01 ± 0.81	46.14 ± 1.34	0.000***	0.000***	0.007**	0.396	0.650
**FM (kg)**	15.52 ± 0.89	49.93 ± 1.94	0.000***	15.84 ± 1.01	14.65 ± 2.02	49.36 ± 1.87	51.24 ± 4.98	0.621	0.000***	0.001***	0.574	0.731
**FFM (kg)**	45.73 ± 2.17	65.90 ± 2.07	0.000***	42.15 ± 1.28	55.55 ± 4.87	60.01 ± 1.56	79.62 ± 2.01	0.175	0.000***	0.000***	0.066.	0.000***
**LM (kg)**	43.41 ± 2.07	62.95 ± 1.79	0.000***	40.01 ± 1.22	52.75 ± 4.63	57.75 ± 1.18	75.09 ± 2.04	0.250	0.000***	0.000***	0.066.	0.000***
**ALM (kg)**	18.27 ± 0.94	28.03 ± 1.24	0.000***	16.71 ± 0.52	22.58 ± 2.13	24.20 ± 0.54	36.97 ± 1.67	0.000***	0.000***	0.007**	0.018*	0.000***
**ALM/W**	0.30 ± 0.01	0.24 ± 0.01	0.000***	0.29 ± 0.00	0.33 ± 0.01	0.22 ± 0.00	0.29 ± 0.01	0.000***	0.000***	0.007**	0.005**	0.000***
**FM/W**	0.26 ± 0.01	0.44 ± 0.01	0.000***	0.28 ± 0.01	0.19 ± 0.02	0.45 ± 0.01	0.42 ± 0.01	0.000***	0.000***	0.007**	0.007**	0.046*
**FFM/H²**	16.94 ± 0.57	24.18 ± 0.43	0.000***	15.83 ± 0.35	19.99 ± 0.50	23.20 ± 0.40	26.45 ± 0.61	0.486	0.000***	0.000***	0.000***	0.000***
**Phase angle 50 MHz°**	5.78 ± 0.19	5.92 ± 0.10	0.464	5.49 ± 0.16	6.58 ± 0.33	5.77 ± 0.10	6.27 ± 0.17	0.106	0.132	0.374	0.005**	0.015*
**Resistance (Ohm)**	659.30 ± 22.35	488.64 ± 10.72	0.000***	696.31 ± 19.70	557.53 ± 21.94	512.15 ± 9.71	433.80 ± 17.41	0.140	0.000***	0.002**	0.002**	0.000***
**Reactance (Ohm)**	64.87 ± 1.40	51.88 ± 0.54	0.000***	65.24 ± 1.70	63.88 ± 2.72	51.97 ± 0.71	51.67 ± 0.79	0.828	0.000***	0.000***	0.683	0.803

ALM, Appendicular Lean Mass; ALM/W, Appendicular Lean Mass to Weight Ratio; BMI, Body Mass Index; CC, Calf circumference; CF, control female; CG, control group; CM, control male; FM, Fat Mass; FM/W, Fat Mass to Weight Ratio; FFM, Fat-Free Mass; FFM/H², Fat-Free Mass Index; LM, Lean Mass; PF, patient female; PG, patient group; PM, patient male; SEM, standard error of the mean; WC, Waist circumference. ***p<0.001; **p<0.01; *p<0.05

Table S2. Dual X-ray absorptiometry (DXA) for body composition (only PG).

	PG	PF	PM	p-value
	**Mean ± SEM**	**Mean ± SEM**	**Mean ± SEM**	**PF vs. PM**
**FM (kg)**	51.7 ± 1.6	53.2 ± 1.9	48.1 ± 3.1	0.164
**FFM (kg)**	56.1 ± 1.4	53.2 ± 0.9	62.9 ± 3.3	0.044*
**LM (kg)**	53.2 ± 1.4	50.3 ± 0.9	59.94 ± 3.5	0.044*
**ALM (kg)**	20.8 ± 0.6	19.7 ± 0.3	23.5 ± 1.4	0.044*
**ALM/W**	0.198 ± 0.006	0.184 ± 0.005	0.23 ± 0.013	0.007**
**FM/W**	0.461 ± 0.012	0.49 ± 0.01	0.396 ± 0.019	0.001**
**FFM/H²**	21.27 ± 0.48	20.24 ± 0.354	23.67 ± 1	0.007**

ALM, Appendicular Lean Mass; ALM/W, Appendicular Lean Mass to Weight Ratio; FFM, Fat-Free Mass; FFM/H^2, Fat-Free Mass Index; FM, Fat Mass; FM/W, Fat Mass to Weight Ratio; LM, Lean Mass; PF, patient female; PG, patient group; PM, patient male; SEM, standard error of the mean. ***$p<0.001$; **$p<0.01$; *$p<0.05$

Table S3. Muscle function.

	CG	PG	p-value	CF	CM	PF	PM	p-values				
	Mean ± SEM	Mean ± SEM	PG vs. CG	Mean ± SEM	Mean ± SEM	Mean ± SEM	Mean ± SEM	Group*Sex	CF vs. PF	CM vs. PM	CF vs. CM	PF vs. PM
**HGS (kg)**	27.35 ± 1.38	29.92 ± 1.88	0.700	25.49 ± 1.19	32.45 ± 2.90	24.89 ± 1.20	41.67 ± 3.12	0.021*	0.750	0.100	0.019*	0.000***
**HGS/W**	0.47 ± 0.02	0.26 ± 0.01	0.000***	0.44 ± 0.02	0.56 ± 0.03	0.23 ± 0.01	0.32 ± 0.02	0.440	0.000***	0.000***	0.004**	0.001**
**HGS/BMI**	1.32 ± 0.09	0.69 ± 0.04	0.000***	1.16 ± 0.05	1.76 ± 0.13	0.60 ± 0.03	0.88 ± 0.06	0.014*	0.000***	0.000***	0.000***	0.000***
**5xCST**	6.09 ± 0.29	10.09 ± 0.54	0.000***	6.49 ± 0.32	5.01 ± 0.15	10.37 ± 0.60	9.44 ± 1.15	0.759	0.000***	0.005**	0.020*	0.438
**TUG (sec)**	5.28 ± 0.28	7.27 ± 0.24	0.000***	5.30 ± 0.29	5.22 ± 0.74	7.45 ± 0.27	6.83 ± 0.45	0.459	0.000***	0.000***	0.028*	0.113
**LS**	1.38 ± 0.03	1.16 ± 0.01	0.000***	1.33 ± 0.04	1.50 ± 0.02	1.14 ± 0.01	1.19 ± 0.03	0.068.	0.000***	0.000***	0.028*	0.114
**SUS**	11.39 ± 0.33	7.35 ± 0.26	0.001**	10.97 ± 0.31	12.56 ± 0.64	7.07 ± 0.30	7.99 ± 0.50	0.588	0.002**	0.195	0.328	0.180
**Stability**	58.73 ± 1.05	50.40 ± 1.58	0.001**	58.09 ± 1.32	60.50 ± 1.32	49.00 ± 1.76	53.67 ± 3.18	0.003**	0.003**	0.187	0.237	0.081.

5xCST, 5 Times Chair Stand Test; CF, control female; CG, control group; CM, control male; HGS, Hand Grip Strength; HGS/BMI, Hand Grip Strength to Body Mass Index Ratio; HGS/W, Hand Grip Strength to Weight Ratio; LS, Leg Strength; PF, patient female; PG, patient group; PM, patient male; SEM, standard error of the mean; SUS, Stand Up Speed; TUG, Timed Up & Go Test. ***p<0.001; **p<0.01; *p<0.05

Table S4. Blood samples, biochemical data.

	CG	PG	p-value	CF	CM	PF	PM	p-values				
	**Mean ± SEM**	**Mean ± SEM**	**PG vs. CG**	**Mean ± SEM**	**Mean ± SEM**	**Mean ± SEM**	**Mean ± SEM**	**Group*Sex**	**CF vs. PF**	**CM vs. PM**	**CF vs. CM**	**PF vs. PM**
**Glucose (mg/dL)**	86.80 ± 1.57	99.43 ± 3.24	0.886	85.64 ± 1.38	90.00 ± 4.56	96.90 ± 4.06	105.33 ± 4.92	0.022*	0.121	0.164	0.239	0.147
**HBA1C (%)**	5.43 ± 0.04	6.07 ± 0.15	0.007**	5.45 ± 0.06	5.40 ± 0.04	5.97 ± 0.18	6.29 ± 0.30	0.043*	0.058.	0.087.	0.786	0.401
**Insulin (mcU/mL)**	7.08 ± 0.77	20.37 ± 1.88	0.000***	7.53 ± 0.97	5.83 ± 1.04	19.10 ± 1.82	23.32 ± 4.65	0.351	0.000***	0.005**	0.347	0.311
**HOMA-IR**	1.51 ± 0.16	5.10 ± 0.52	0.000***	1.59 ± 0.20	1.31 ± 0.26	4.65 ± 0.51	6.15 ± 1.26	0.277	0.000***	0.005**	0.456	0.195
**Cr (mg/dL)**	0.84 ± 0.04	0.83 ± 0.03	0.718	0.79 ± 0.04	0.96 ± 0.09	0.77 ± 0.03	0.97 ± 0.07	0.746	0.493	0.740	0.593	0.684
**GFR (ml/min)**	85.67 ± 3.58	87.40 ± 2.72	0.709	84.45 ± 4.02	89.00 ± 8.40	88.14 ± 3.21	85.67 ± 5.38	0.854	0.487	0.757	0.514	0.667
**GOT (U/L)**	18.87 ± 0.82	18.63 ± 0.63	0.761	17.91 ± 0.56	21.50 ± 2.40	18.52 ± 0.75	18.89 ± 1.23	0.180	0.588	0.303	0.232	0.796
**GPT (U/L)**	17.40 ± 1.59	25.53 ± 1.70	0.002**	16.64 ± 1.97	19.50 ± 2.60	24.05 ± 1.82	29.00 ± 3.67	0.008**	0.008**	0.075.	0.265	0.286
**ALP (U/L)**	70.40 ± 5.42	79.67 ± 4.48	0.306	71.18 ± 6.92	68.25 ± 8.50	81.76 ± 5.98	74.78 ± 5.39	0.779	0.283	0.522	0.821	0.484
**GGT (U/L)**	20.73 ± 2.08	32.20 ± 3.81	0.054.	20.55 ± 2.78	21.25 ± 2.17	27.14 ± 3.93	44.00 ± 7.77	0.026*	0.283	0.025*	0.744	0.023*
**CHOL (mg/dL)**	214.53 ± 6.24	172.98 ± 7.04	0.000***	215.82 ± 7.85	211.00 ± 10.54	182.10 ± 7.64	151.72 ± 13.35	0.371	0.009**	0.019*	0.746	0.046*
**TG (mg/dL)**	72.80 ± 8.49	121.20 ± 5.69	0.000***	71.91 ± 11.27	75.25 ± 9.72	118.38 ± 5.66	127.78 ± 14.00	0.760	0.000***	0.039*	0.869	0.459
**HDL (mg/dL)**	80.00 ± 4.48	46.40 ± 1.78	0.000***	81.73 ± 5.50	75.25 ± 7.98	48.19 ± 2.21	42.22 ± 2.59	0.887	0.000***	0.000***	0.543	0.126
**LDL (mg/dL)**	119.97 ± 6.94	98.12 ± 6.18	0.035*	119.71 ± 9.05	120.70 ± 9.53	103.50 ± 6.21	85.57 ± 14.45	0.494	0.143	0.155	0.952	0.189
**PTH (pg/ml)**	50.47 ± 3.14	60.00 ± 2.96	0.027*	49.45 ± 3.35	53.25 ± 8.14	60.24 ± 3.06	59.44 ± 7.18	0.721	0.035*	0.621	0.611	0.905
CTX	0.60 ± 0.04	0.31 ± 0.03	0.000***	0.64 ± 0.04	0.49 ± 0.05	0.32 ± 0.03	0.28 ± 0.05	0.326	0.000***	0.028*	0.064.	0.454
**OC**	28.26 ± 1.98	17.85 ± 1.07	0.000***	29.54 ± 1.95	24.75 ± 5.30	18.57 ± 1.19	16.16 ± 2.27	0.001***	0.000***	0.076.	0.647	0.205
**25OHD (ng/mL)**	29.27 ± 2.93	22.27 ± 2.03	0.055.	29.18 ± 4.00	29.50 ± 2.10	23.62 ± 2.28	19.11 ± 4.20	0.423	0.202	0.143	0.964	0.317
**Ca (mg/dL)**	9.45 ± 0.05	9.42 ± 0.07	0.333	9.43 ± 0.06	9.50 ± 0.07	9.42 ± 0.09	9.41 ± 0.13	0.729	0.347	0.698	0.248	0.909
**P (mg/dL)**	3.75 ± 0.08	3.56 ± 0.11	0.197	3.84 ± 0.09	3.48 ± 0.11	3.68 ± 0.11	3.27 ± 0.26	0.034*	0.500	0.189	0.077.	0.032*
**Mg (mg/dL)**	2.06 ± 0.03	2.01 ± 0.03	0.250	2.06 ± 0.04	2.07 ± 0.04	2.04 ± 0.04	1.93 ± 0.03	0.287	0.762	0.019*	0.832	0.033*
**ALB (mg/dL)**	4.62 ± 0.03	4.58 ± 0.05	0.516	4.59 ± 0.04	4.71 ± 0.06	4.56 ± 0.05	4.63 ± 0.12	0.730	0.709	0.678	0.139	0.623
**Pre-ALB (mg/dL)**	27.70 ± 1.14	25.90 ± 0.71	0.170	26.56 ± 1.38	30.83 ± 0.89	25.85 ± 0.88	26.02 ± 1.26	0.196	0.653	0.036*	0.099.	0.913
**RBP (mg/dL)**	5.32 ± 0.34	4.86 ± 0.14	0.233	5.12 ± 0.45	5.88 ± 0.20	4.86 ± 0.16	4.86 ± 0.26	0.163	0.706	0.024*	0.472	0.856
**CRP (mg/L)**	0.97 ± 0.13	4.52 ± 0.64	0.000***	0.91 ± 0.15	1.13 ± 0.29	4.47 ± 0.80	4.64 ± 1.06	0.001**	0.001***	0.054.	0.448	0.572

25OHD, 25-Hydroxyvitamin D; ALB, Albumin; ALP, Alkaline Phosphatase; Ca, Calcium; CF, Control Female; CG, Control group; CHOL, Cholesterol; CM, Control Male; Cr, Creatinine; CRP, C-Reactive Protein; CTX, C-terminal telopeptide of type I collagen; GFR, Glomerular Filtration Rate; GGT, Gamma-Glutamyl Transferase; Glucose, Glucose; GOT, Glutamic Oxaloacetic Transaminase; GPT, Glutamic Pyruvic Transaminase; HBA1C, Hemoglobin A1c; HDL, High-Density Lipoprotein Cholesterol; HOMA-IR, Homeostatic Model Assessment for Insulin Resistance; Insulin, Insulin; LDL, Low-Density Lipoprotein Cholesterol; Mg, Magnesium; OC, Osteocalcin; P, Phosphorus; PF, Patient Female; PG, Patient Group; PM, Patient Male; Pre-ALB, Prealbumin; PTH, Parathyroid Hormone; RBP, Retinol Binding Protein; SEM, standard error of the mean; TG, Triglycerides. ***$p<0.001$; **$p<0.01$; *$p<0.05$.
